# Classes of depression symptom trajectories in patients with major depression receiving a collaborative care intervention

**DOI:** 10.1371/journal.pone.0202245

**Published:** 2018-09-07

**Authors:** Juliana J. Petersen, Johannes Hartig, Michael A. Paulitsch, Manuel Pagitz, Karola Mergenthal, Sandra Rauck, Andreas Reif, Ferdinand M. Gerlach, Jochen Gensichen

**Affiliations:** 1 Institute of General Practice, Goethe-University Frankfurt am Main, Frankfurt am Main, Germany; 2 Department of Educational Quality and Evaluation, German Institute for International Educational Research, Frankfurt am Main, Germany; 3 Department of Psychiatry, Psychosomatic Medicine and Psychotherapy, University Hospital Frankfurt, Frankfurt, Germany; 4 Institute of General Practice and Family Medicine, Ludwig-Maximilians University Clinic, Munich, Germany; PLOS, UNITED STATES

## Abstract

**Purpose:**

Collaborative care is effective in improving symptoms of patients with depression. The aims of this study were to characterize symptom trajectories in patients with major depression during one year of collaborative care and to explore associations between baseline characteristics and symptom trajectories.

**Methods:**

We conducted a cluster-randomized controlled trial in primary care. The collaborative care intervention comprised case management and behavioral activation. We used the Patient Health Questionnaire-9 (PHQ-9) to assess symptom severity as the primary outcome. Statistical analyses comprised latent growth mixture modeling and a hierarchical binary logistic regression model.

**Results:**

We included 74 practices and 626 patients (310 intervention and 316 control recipients) at baseline. Based on a minimum of 12 measurement points for each intervention recipient, we identified two latent trajectories, which we labeled ‘fast improvers’ (60.5%) and ‘slow improvers’ (39.5%). At all measurements after baseline, ‘fast improvers’ presented higher PHQ mean values than ‘slow improvers’. At baseline, ‘fast improvers’ presented fewer physical conditions, higher health-related quality of life, and had made fewer suicide attempts in their history.

**Conclusions:**

A notable proportion of 39.5% of patients improved only ‘slowly’ and probably needed more intense treatment. The third follow-up in month two could well be a sensible time to adjust treatment to support ‘slow improvers’.

## Introduction

Depression is a leading cause of disease burden [1], and it is estimated that up to 90% of patients with depression are treated in primary care [2]. ‘Collaborative care’ is a complex strategy aimed at improving care of such patients. While the individual elements of collaborative care interventions vary, they typically include a multi-professional team approach, structured management plans, scheduled follow-ups, and enhanced inter-professional communication [3,4]. Usually a case manager works in conjunction with the primary care physician, often with the support of a mental health specialist. Evidence shows that collaborative care is effective in reducing depression symptoms, improving patient satisfaction, and enhancing mental health quality of life [4]. It is effective for people with depression alone, as well as for those with additional multiple chronic physical conditions [5].

In the United States, efforts have been undertaken to implement collaborative care interventions (e.g., [6]). In European countries, an increasing number of trials has been undertaken in recent years to assess how collaborative care interventions can be adapted to be effective in specific health care settings [7].

An increasing body of evidence on their effectiveness highlights the need to understand in detail the way in which these complex interventions work. A recently published review, however, found that international guidelines on depression provide too little practical advice on the organization and maintenance of collaborative care [8].

For primary care physicians, it is difficult to predict the course of a patient’s illness over time. Symptom trajectories in patients with depression are heterogeneous; some remit with or without medical intervention, whilst others experience varying symptoms with differing degrees of intensity, or the course of the disease even becomes chronic.

With the development of group-based trajectory modeling, statistical methodologies for analyzing trajectories have advanced substantially over the last 15 years. These methods allow the identification and characterization of distinct subgroups of patients with similar trajectory patterns [9].

Trajectory classes depend upon the analyzed samples. While most studies on trajectory modeling have been conducted on general population samples [10], others have used specific population samples, such as patients with breast cancer [11]. Few studies have recruited primary care patients: Gunn et al., for instance, analyzed depressive symptom trajectories in primary care patients in Australia and found five distinct trajectories of depressive symptoms [12]. Other studies have analyzed symptom trajectories in patients with major depressive disorder. The Sequenced Treatment Alternatives to Relieve Depression (STAR*D) trial, for instance, analyzed symptom trajectories in outpatients with major depressive disorder who had received citalopram for up to 14 weeks. The authors found that the trajectories of all depressive symptoms differed significantly between remitters and non-remitters [13].

Up to now, little is known about the symptom trajectories of collaborative care recipients with depression. Information on trajectories would allow interventions to be further optimized by enabling patients who are at greater risk of symptom persistence or deterioration to be identified. It would then be easier to evaluate more accurately when a patient’s therapy should be intensified.

The aims of the study were therefore 1) to characterize symptom trajectories in patients with major depression during one year of primary care-based collaborative depression care and 2) to assess associations between patients’ baseline characteristics and symptom trajectories.

## Methods

### Study design and participants

The cluster-randomized controlled ‘Primary care monitoring for depressive patients trial’ (PRoMPT) was conducted on the effectiveness of a collaborative care intervention for patients with major depression in 74 general practices in the federal state of Hesse, Germany, between 2005 and 2008. The data safety and monitoring board stratified the practices according to the size of the city in which the practices were located and performed computer-based randomization. Patients, healthcare assistants (HCAs, ‘Medizinische Fachangestellte’), general practitioners (GPs), and researchers were not blinded to assignment after completion of the baseline assessment because of the practice staff training required for the behavioral intervention. Details on the methods employed in the trial [14] have been published elsewhere. The trial was registered at Current Controlled Trials (ISRCTN66386086). The inclusion criteria for patients were diagnosis of major depression with indication for any antidepressive treatment, aged 18 to 80, access to private telephone, ability to give informed consent and communicate in German. The diagnosis of major depression was based on a PHQ-9 score of more than nine points, and ‘categorical criteria’ in the PHQ-9, i.e., five or more of the nine depressive symptom criteria were present on “more than half the days” in the previous two weeks, and one of the symptoms was depressed mood or anhedonia. One of the symptom criteria (“thoughts that you would be better off dead or of hurting yourself in some way”) counted regardless of duration [15]. The diagnosis was confirmed by the general practitioner. Exclusion criteria were confirmed pregnancy, severe alcohol or illicit drug consumption and acute suicidal ideation as assessed by the GP. We used written consent procedures, and all participants gave their informed consent. The institutional review board of Goethe-University Frankfurt am Main approved the study protocol. Main results on depression symptoms at 12 months [16] and 24 months have been published elsewhere [17,18].

### Intervention

The intervention lasted 12 months (between baseline and the 12-month assessment). We designed our case management intervention in accordance with the Chronic Care Model, which emphasizes the need for care to be planned, proactive and patient-centered [19]. The model identifies key elements of high-quality care provision for patients with chronic illnesses, i.e. self-management support, provision of clinical information systems, delivery system redesign, and decision support. One GP and one healthcare assistant from each practice were assigned to the intervention group and were responsible for performing the intervention. In Germany, GP practices generally employ one or more healthcare assistants. Their role is comparable to healthcare assistants in the UK and to medical assistants in the United States [20]. They perform basic clinical tasks such as intramuscular injections and ECGs. The required qualifications in Germany are three years of vocational (on-the-job) training that includes one and a half days of school per week.

One healthcare assistant from each practice received interactive training in depression, communication skills, telephone monitoring, and behavioral activation for the patient. In accordance with our study protocol, HCAs were required to contact patients biweekly in the first six weeks and monthly thereafter. Thus, the recommendation was to monitor patients a total of 15 times after baseline (weeks 2, 4, 6, 10, 14, 18, 22, 26, 30, 34, 38, 42, 46, 50 and 54). They monitored symptoms and adherence to medication using a structured questionnaire called the ‘Depression-Monitoring-List’ (DeMoL) [21] and encouraged patients to perform self-management activities (behavioral activation), such as participation in pleasant and/or social activities and medication adherence. The assistants provided information to the GP in a structured report that stratified the urgency of the contact in accordance with symptom severity. This intervention was provided in addition to usual care. The mean duration of these interviews was 12 minutes.

### Data collection

Data collection in intervention and control groups consisted of three assessments (at baseline, after 6 and 12 months) by means of self-rating questionnaires for patients, as well as case report forms for GPs. The questionnaire contained the validated German version of the Patient Health Questionnaire (PHQ-9) to assess depressive symptoms, the primary outcome. The PHQ-9 consists of nine items, each of which is scored on a rating-scale of 0 (not at all) to 3 (nearly every day), with a total sum score that ranges from 0 to 27 (high scores indicate more severe depression) [15]. Health-related quality of life was measured by means of the EuroQol (EQ-5D) [22]. The EQ-5D is a generic instrument that measures health-related quality of life using a visual analogue scale (range, 0 to 100; higher ratings indicate higher quality of life). Number of physical comorbid conditions was assessed by counting the documented diagnoses from different diagnostic groups (excluding all psychiatric diagnoses) in the patient records.

As mentioned above, healthcare assistants also used the ‘Depression-Monitoring-List’, for symptom monitoring in the intervention group. The DeMoL consists of 12 items. The first nine items correspond to the validated Patient Health Questionnaire (PHQ-9) for the assessment of depression symptoms. The other three items relate to medication adherence (“Since our last discussion, have you taken the medication as agreed with the GP?”), achievement of personal treatment goals, and an open question on whether the patient has anything else of relevance that he/she would like to tell the GP practice team. We used the PHQ-9 monitoring data to analyze symptom trajectories in intervention recipients.

### Statistical analysis

For the descriptive analyses, we calculated mean value, standard deviation and the frequency distributions of the response categories. We identified latent subgroups (‘classes’) of depression trajectories using growth mixture modeling [9]. Latent growth mixture modeling combines a person-oriented approach with traditional variable-oriented growth curve modeling, permitting continuous latent growth factors (i.e., intercept and slope) to be related to time. It uses categorical latent variables to identify different classes of trajectories within a group. The number of latent trajectories was chosen according to the lowest Bayesian Information Criterion (BIC) along with size and interpretation of the classes. We assessed two- to four-class models.

At the data analysis stage, we decided to restrict the analyses to patients who received 12 or more contacts, because we did not want the sample in this analysis to be too heterogeneous. In fact, growth mixture modeling does not take into account the exact time span between intra-individual monitoring contacts. That means we could not have said whether a patient that, for example, was monitored only 5 times in total, was monitored as required by the study protocol previous to dropping out, or whether he remained in the study until the end but was visited at far greater intervals than foreseen in the protocol.

Due to the multilevel structure of the data, we used hierarchical regression models to compare baseline characteristics of classes, taking into account patient observations (level 1), nested within general practices (level 2). We constructed the hierarchical binary logistic regression model in several steps. In a first step, we calculated a ‘null’ model by including practices as an independent variable to assess the Intraclass Correlation Coefficient (ICC) for binary data. In a second step, fixed independent level 1 variables were added and tested for their association with the dependent variable, the latent trajectory class. The estimation method was the Laplace approximation. Results for each coefficient are presented as b-coefficients (with their confidence intervals), p-values for the Wald-statistic, and odds ratios with confidence intervals. All p values were reported as being statistically significant on the basis of a significance level of 0.05. We used Mplus7 (version 7.4) for growth mixture modeling [9,23], R-package lme4 (version 1.1–13) for the hierarchical regression model [24] and the IBM SPSS (Statistical Package for Social Sciences) Statistics for Windows, version 22.0, Armonk, NY: IBM Corp [25] for the remaining analyses.

## Results

### Study population

**Fig 1:**
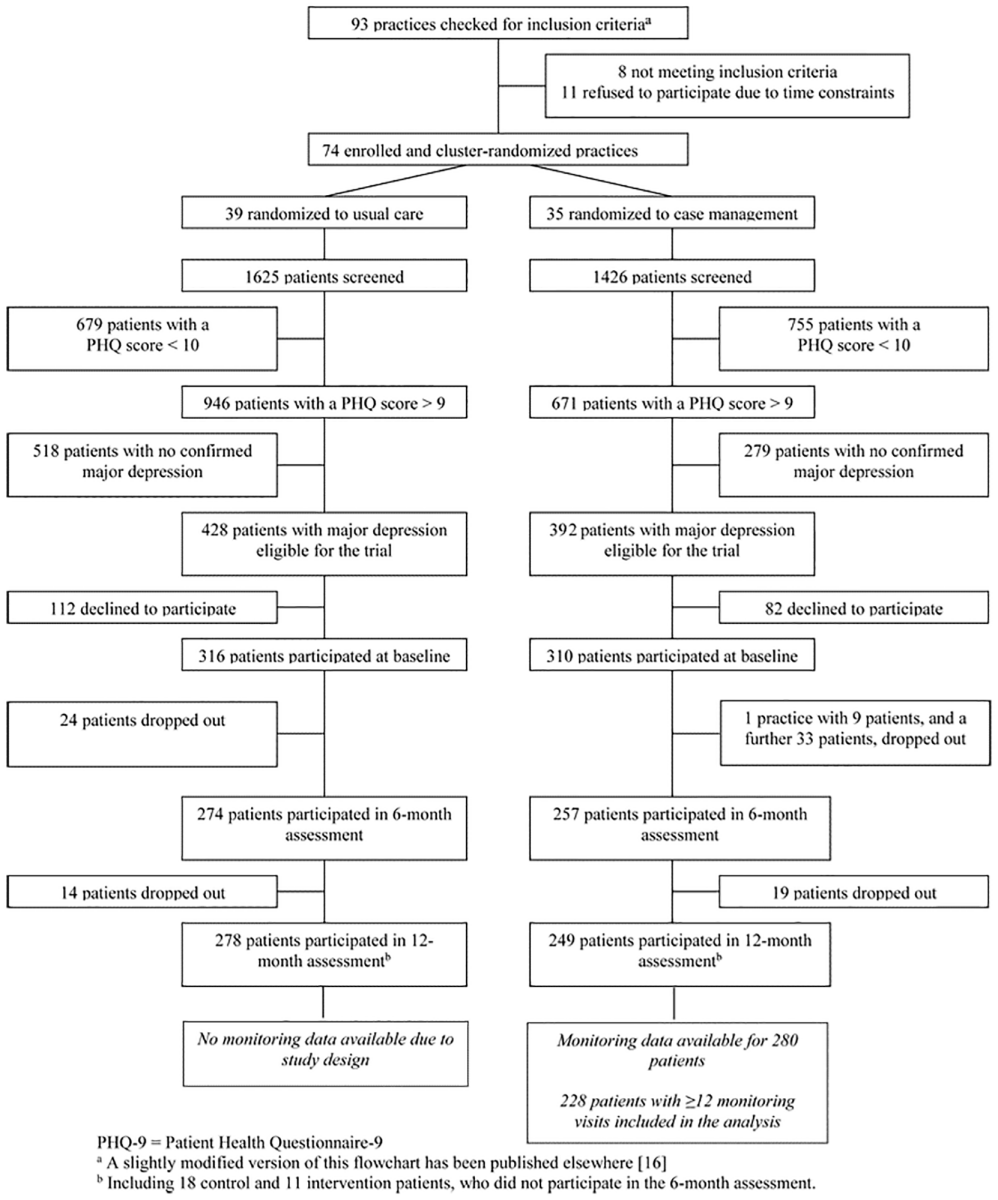
Flow diagram of study participants. PHQ-9 = Patient Health Questionnaire-9. ^a^ A slightly modified version of this flowchart has been published elsewhere [16]. ^b^ Including 18 control and 11 intervention patients, who did not participate in the 6-month assessment.

Details on the practice and patient recruitment process and baseline description have already been published elsewhere [16,17,26]. In brief, we checked 93 practices to recruit the required sample of 74 practices and 74 practice teams (each consisting of one GP and one healthcare assistant) (Fig 1). The practice teams referred 3051 patients to the study for screening. A diagnosis of major depression was confirmed for 820 patients (428 control patients and 392 intervention recipients) based on the PHQ-9 score, additional categorical criteria in the PHQ-9, and a structured clinical interview with the GP. We included 626 patients (310 intervention and 316 control recipients) at baseline (Fig 1) [16].

We enrolled a mean of 8.1 patients (SD, 2.7) from each control group practice and 8.9 patients (SD, 2.6) from each intervention group practice. After 12 months, it was possible to assess 249 (80.3%) patients from the intervention and 278 (88.0%) from the control group [16]. Monitoring data was available for 280 (90.3%) intervention recipients. Of these, n = 228 received between 12 and 16 monitoring contacts, with a peak at 15, as specified in the study protocol (see S1 Fig).

Most practices (100% in the intervention group vs. 92.3% in the control group) were self-owned by one or two physicians, and one-third (32.4% vs. 46.2%) were located in a rural area. The practices had been run by their primary care physician owners for a mean of 13.4 years (SD, 9.3) in the intervention group and 10.7 years (SD, 7.7) in the control group [16]. The main socio-demographic characteristics, such as age, sex, marital and employment status ascertained at baseline were similar for both patient groups (Table 1) [16]. The mean PHQ-9 depression score was 17.3 (SD, 3.6) in the intervention and 17.3 (SD, 3.5) in the control group.

**Table 1:** Socio-demographic and clinical characteristics of participants at baseline^a^.

	Intervention recipients	Control recipients
*Sociodemographic characteristics*		
Age		
Age, *mean (SD)*, *y*	51.2 (14.6)	50.3 (14.7)
Age ≥ 65 years, *n (%)*	61 (19.7)	54 (17.2)
Female, *n (%)*	228 (73.6)	245 (77.5)
Civil status		
Married, *n (%)*	173 (55.8)	160 (50.6)
Single, *n (%)*	62 (20.0)	74 (23.4)
Divorced, *n (%)*	46 (14.8)	43 (13.6)
Widowed, *n (%)*	27 (8.7)	37 (11.7)
Unknown, *n (%)*	2 (0.6)	2 (0.6)
Employment status		
Employed (full- or part-time), *n (%)*	133 (42.9)	147 (46.5)
Unemployed, *n (%)*	42 (13.5)	40 (12.7)
Retired, *n (%)*	65 (21.0)	60 (19.0)
Housewife/-husband, *n (%)*	30 (9.7)	24 (7.6)
Other (e.g., vocational training), *n (%)*	38 (12.3)	41 (13.0)
Unknown, *n (%)*	2 (0.6)	4 (1.3)
*Clinical characteristics*		
Depression, *mean PHQ-9 (SD)*	17.3 (3.6)	17.3 (3.5)
PHQ sum score		
Mild or moderate (sum score ≤14), *n (%)*	82 (26.5)	75 (23.9)
Moderately severe (sum score 15–19), *n (%)*	147 (47.6)	159 (50.6)
Severe (sum score ≥ 20), *n (%)*	80 (25.9)	80 (25.5)
Duration of current depressive episode, *mean (SD)*, *weeks*	17.5 (23.3)	21 (28.5)
Receiving maintenance treatment, *n (%)*^b^	232 (74.8)	234 (74.5)
Number of physical comorbid conditions, *mean (SD)*^c^	3 (2.2)	3 (2.3)
Health-related quality of life, mean EQ-5D (SD)	61.1 (20.2)	59.8 (21.3)

^a^ Analyses based on n (max) = 310 intervention recipients and n (max) = 316 control recipients.

^b^ Defined as patients receiving depression treatment (depression diagnosis known by the GP before starting the trial).

^c^ Number of physical comorbid conditions were assessed by counting the documented diagnoses of different diagnostic groups (excluding all psychiatric diagnoses) in the patient records.

### Trajectory classes

**Fig 2:**
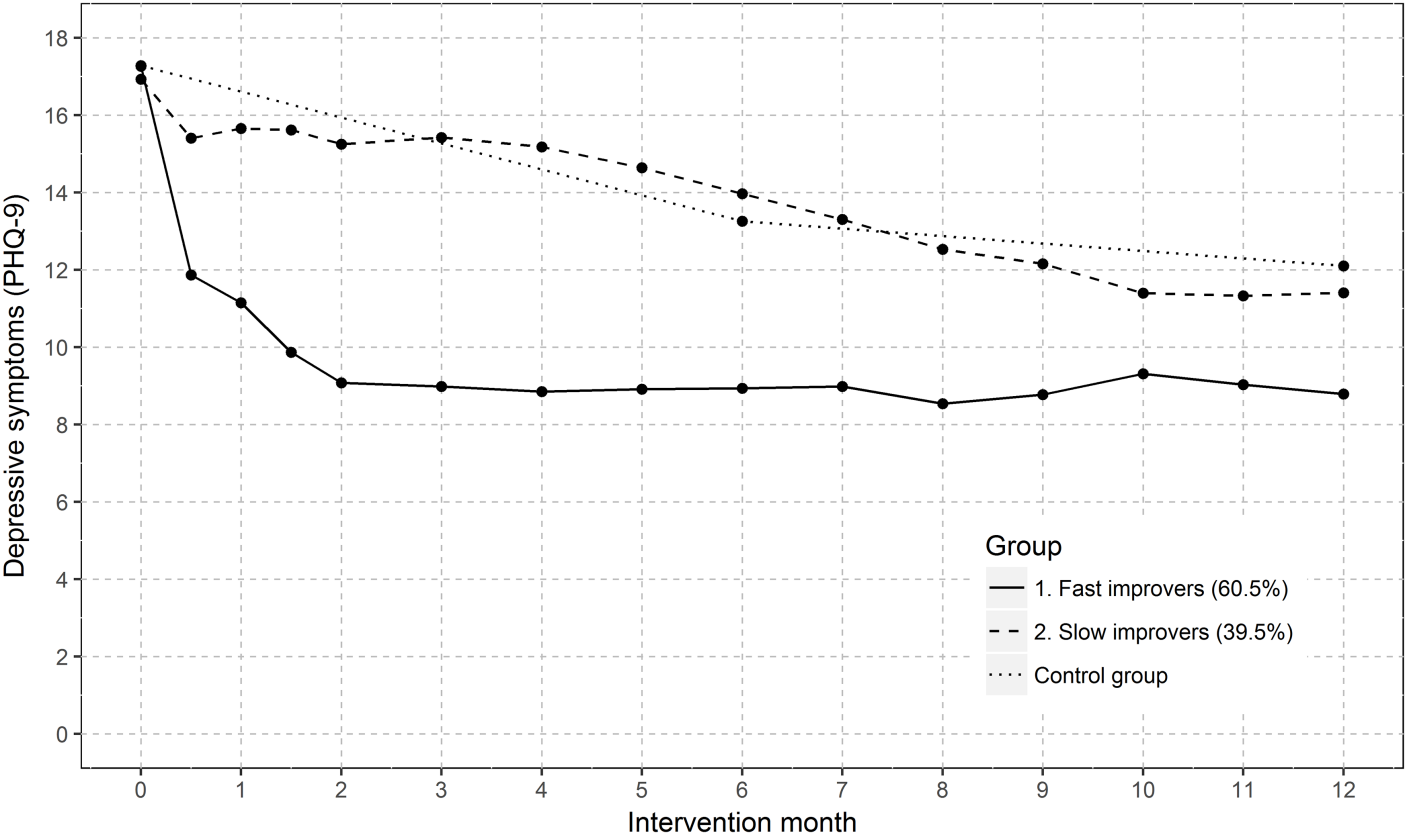
Trajectories of depressive symptoms (PHQ-9) over 12 intervention months for the best fitting two-class model. 228 patients with ≥ 12 PHQ measurements were included in analyses.

According to the BIC, a growth mixture model with four latent classes provided the best fit (BIC _2 classes_ = 20284; BIC _3 classes_ = 20256; BIC _4 classes_ = 20249; BIC _5 classes_ = 20259). However, as groups in solutions with more than two classes became small and were not clearly distinguishable from one another, the two-class solution provided the best separation and interpretation of groups. Two patient subgroups with distinct latent trajectory classes were identified (Fig 2), i.e. a relatively ‘fast’ improving class (60.5% of the sample) and a ‘slowly’ improving class (39.5% of the sample).

We also conducted growth mixture modeling for all the patients for whom PHQ data was available (see S2 Fig). As can be seen, these results also show a two-class solution and therefore do not differ significantly from the results based on patients with ≥ 12 PHQ measurements. ‘Slow improvers’ presented worse PHQ-9 mean values at each measurement point after baseline, and the values differed statistically significantly from those of ‘fast’ improvers. After 12 months, their PHQ mean values were also worse than those of ‘fast improvers’.

The ‘null’ model, which included only practices as an independent variable, yielded an Intraclass Correlation Coefficient for binary data of 0.1045 (10.45%). Table 2 displays the results of the final hierarchical binary logistic regression model. Patients with a history of suicide attempts (OR 2.778 [1.122; 6.876]), with more physical conditions (OR 1.181 [1.001; 1.393]), and lower health-related quality of life (OR 0.973 [0.956; 0.991]) were more likely to be ‘slow improvers’. The two groups did not differ at baseline in terms of age, sex, education, marital status, depression severity and start of treatment.

**Table 2:** Comparison of baseline characteristics of the two groups ‘fast’ and ‘slow’ improvers^a^.

		Fast improvers	Slow improvers	b-coefficient	95% CI (b-coefficients)	p-value (Wald)	Odds ratio	95% CI (odds ratio)
*Socio-demographic characteristics*								
Intercept				1.085	-1.956–4.126	0.484	2.96	0.141–61.943
Age (in years), mean (SD)		52.2 (15.1)	52.2 (13.6)	-0.001	-0.028–0.026	0.952	0.999	0.973–1.027
Sex	male	40 (28.6)	18 (20.5)	-0.648	-1.506–0.21	0.139	0.523	0.222–1.234
	female (reference)	100 (71.4)	70 (79.5)					
Education	higher education entrance qualification	24 (17.3)	25 (28.7)	0.414	-0.502–1.329	0.376	1.512	0.606–3.778
	no higher education entrance qualification (reference)	115 (82.7)	62 (71.3)					
Civil status	married	80 (57.1)	43 (48.9)	0.089	-0.627–0.805	0.807	1.093	0.534–2.237
	not married (reference)	60 (42.9)	45 (51.1)					
*Clinical characteristics*								
Depression, mean PHQ-9 (SD)		17.2 (3.6)	16.9 (3.6)	-0.043	-0.149–0.063	0.429	0.958	0.861–1.066
Duration of current depressive episode (in weeks), mean (SD)		19.1 (23.7)	17.8 (23.4)	0.001	-0.016–0.018	0.896	1.001	0.984–1.019
Therapy	maintenance therapy^b^	105 (75)	70 (79.5)	0.029	-0.816–0.873	0.947	1.029	0.442–2.394
	treatment starter (reference)	35 (25)	18 (20.5)					
History of suicide attempt(s)	yes	19 (13.7)	25 (28.4)	1.022	0.115–1.928	0.027	2.778	1.122–6.876
	no (reference)	120 (86.3)	63 (71.6)					
Health related quality of life, mean EQ-5D (SD)		65 (1.9)	57.2 (20)	-0.027	-0.045 –-0.009	0.003	0.973	0.956–0.991
Number of physical comorbid conditions^c^		3.06 (1.9)	3.6 (2.8)	0.166	0.001–0.331	0.049	1.181	1.001–1.393

^a^ Based on results of hierarchical binary logistic regression model

^b^ Defined as patients receiving depression treatment (depression diagnosis known by the GP before starting the trial)

^c^ Number of physical comorbid conditions were assessed by counting the documented diagnoses of different diagnostic groups (excluding all psychiatric diagnoses) in the patient records.

## Discussion

To our knowledge, this was the first study to explore the symptom trajectory classes of patients with major depression receiving a one-year collaborative care intervention in German GP practices. Using growth mixture modeling, and the PHQ-9 as a continuous measure, we identified two latent trajectory classes of depressive symptoms in these patients, which we labeled ‘fast’ and ‘slow’ improvers.

The finding that approximately 60% of patients improved relatively fast is reassuring because it indicates that the intervention benefits most patients. Our findings suggest that in primary care, health care assistants without specialized training in mental health care can help patients with depression by regularly monitoring symptoms and providing behavioral activation. Numbering more than 500,000, HCAs represent one of the biggest professional health care groups in Germany [27].

The effect size was small but similar to that in other case management trials on depression: A meta-analysis published by Coventry et al. found that–compared to usual care–collaborative care is associated with improvements in depressive symptoms with a standardized mean difference of -0.28, 95%CI -0.33 to 0.23 [28]. We did not expect greater effects because the intervention was performed in addition to usual care. We designed our intervention to manage patients with depression without making excessive demands on the limited resources of the small primary care practices that are mainly responsible for treating them. The COBRA (Cost and Outcome of Behavioural Activation versus Cognitive Behavioural Therapy for Depression) trial identified behavioral activation delivered by junior mental health workers–supervised by experienced specialists—as non-inferior to cognitive behavioral therapy in reducing depression symptoms at 12 months [29]. The COBRA study has been criticized for relying on a supervision system that is far removed from real world scenarios [30]. In contrast, this trial’s intervention was embedded in a real-life setting, and did not require the creation of either new interfaces or the involvement of professions outside the family practice.

A notable proportion of 39.5% of patients improved only ‘slowly’. Strikingly, the symptom trajectory of this subgroup was similar to that of the control group, indicating that treatment intensification for these patients is probably necessary. The symptom plateau of ‘fast improvers’, however, which starts at about month two and continues until month 12, may be indicative of a need for intensification in these patients too. Since our intervention was complex, we were unable to identify which element(s) of the treatment should be intensified. Further research will be necessary to find out exactly how this should be realized, whereby specialist psychiatric services should possibly be involved earlier. An ongoing study in Germany is evaluating the effects of a primary care-based depression care intervention with caseload supervision for case managers (health care assistants) by a psychiatric specialist [31]. Furthermore, collaborative depression care should incorporate treatment strategies that prevent relapse.

The number of classes identified in this study was lower than the number identified in earlier cohort studies conducted in Dutch and Australian primary care patients, which both identified five-class models [12,32]. The disparity is probably because in our study group solutions with more than two classes became too small and were not clearly distinguishable from one another (we acknowledge this to be a limitation of this study). However, since each of the two classes summarized two classes in the 4-class solution relatively clearly, we think that the two models can be interpreted similarly.

Our findings suggest that subjects with major depression who have a history of suicidal ideation, additional physical conditions, and low self-rated, health-related quality of life, warrant more treatment attention because they are at higher risk of chronicity. Similarly, Gunn et al. found that members of the ‘severe’ trajectory group in their study were more likely to have chronic illnesses, disabilities, and lower self-rated health [12].

In a collaborative care intervention for typical primary care patients with multiple conditions, depression symptoms and quality of life could be improved by modifying specific patient and clinician behaviors (close monitoring of disease control parameters and timely treatment adjustments at an early follow-up of two months) [33].

### Strengths and limitations

One strength of this study is the high number of symptom measurements per intervention recipient and the short intervals between them. Group-based trajectory models require a large sample size and a high number of assessments [10]. As this is difficult and costly to achieve, most longitudinal studies rely on longer intervals [10]. When comparing intervention and control groups in this study it should be taken into account that the trajectories of control recipients were based on only three measurements, whereas the trajectory courses of intervention recipients were based on at least 12, and could therefore be estimated more accurately.

This study has several limitations: methodological limitations meant we were unable to include medication changes over time in the statistical model. Post-hoc analyses of clinical trials suggest that a drug non-response can already be observed within the first 14 days of treatment, and that this poor outcome is predictive of treatment outcome after 6–8 weeks [34,35]. However, the two-step switch/augmentation strategy used in the Early Medication Change (EMC) trial for this risk group was no more effective than the control strategy [36].

A further limitation is that a notable percentage of loss to follow-up occurred at 12 months. However, previously conducted sensitivity analyses have shown that the effects of the intervention on depression symptoms after 12 months remain statistically significant and stable under unfavorable assumptions about participation in follow-up assessments [16].

The results of this study can only be generalized to intervention patients that have had ≥12 monitoring visits. However, as in our study, a lower number of visits may result from patients dropping out during the monitoring period (after receiving a regular monitoring schedule), or when patients who remain until the end of the study are visited at longer intervals. It will be possible to address this issue in an upcoming evaluation study, as it has been announced that an intervention based on the one presented here will be implemented as routine care for 2000 patients in GP practices in the federal state of Hesse. The intervention will be funded by a so-called ‘innovation fund” (“Innovationsfonds”) and conducted by a large health insurance fund (“Techniker Krankenkasse”) in cooperation with the Hesse Association of Statutory Health Insurance Physicians and other institutions [37]. The large sample size will allow researchers to describe symptom courses of all patients more accurately.

## Conclusions

As the course taken by depressive symptoms is heterogeneous, the trajectory model provides helpful information on how to stratify patients who may be at risk of not recovering significantly during the collaborative care intervention. These patients probably need more intense treatment at certain points during the one-year intervention. In our study, the third follow-up in month two appeared to be a sensible time to adjust treatment, as it is reasonable to assume that if patients have not improved after the third or fourth contact, they will require more intense therapy.

Since this was the first trial on collaborative care for patients with depression receiving treatment in GP practices in Germany, we recommend that future collaborative intervention studies involving similar interventions should build upon these findings and assess ways to intensify effectively therapy for these patients.

## Supporting information

S1 Fig Frequency of HCA contacts per patient. (TIF)

S2 Fig Trajectories of depressive symptoms (PHQ-9) over 12 intervention months for the best fitting two-class model. All 280 patients for whom monitoring data was available were included in the analyses.
(TIF)
