# Supplementary figures and images for: Classes of depression symptom trajectories in patients with major depression receiving a collaborative care intervention
Source: PLoS One. 2018 Sep 7;13(9):e0202245. doi: 10.1371/journal.pone.0202245 (PMC6128457; doi:10.1371/journal.pone.0202245)

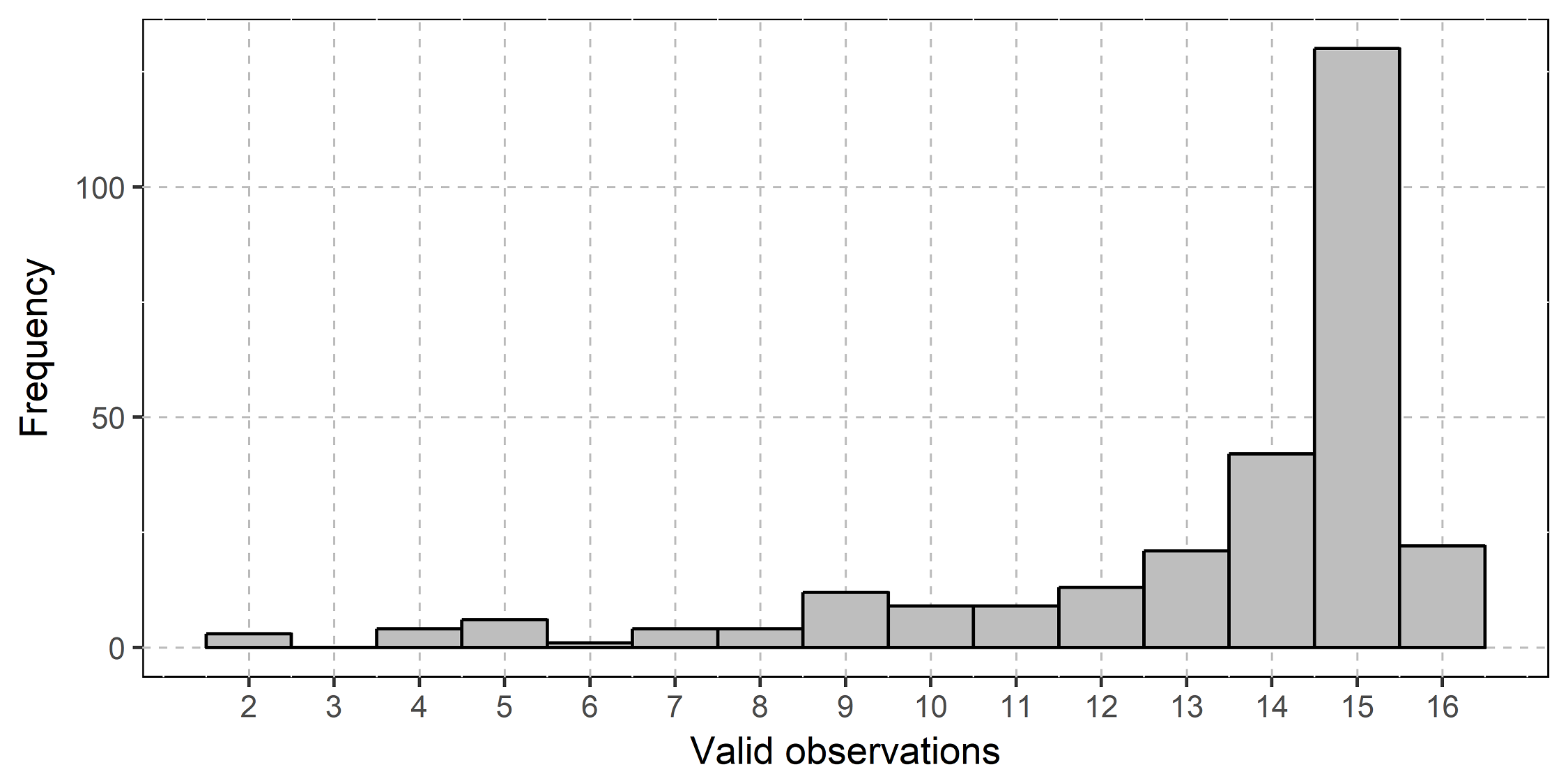

Supplement: S1 Fig — (TIF) [file pone.0202245.s001.tif]

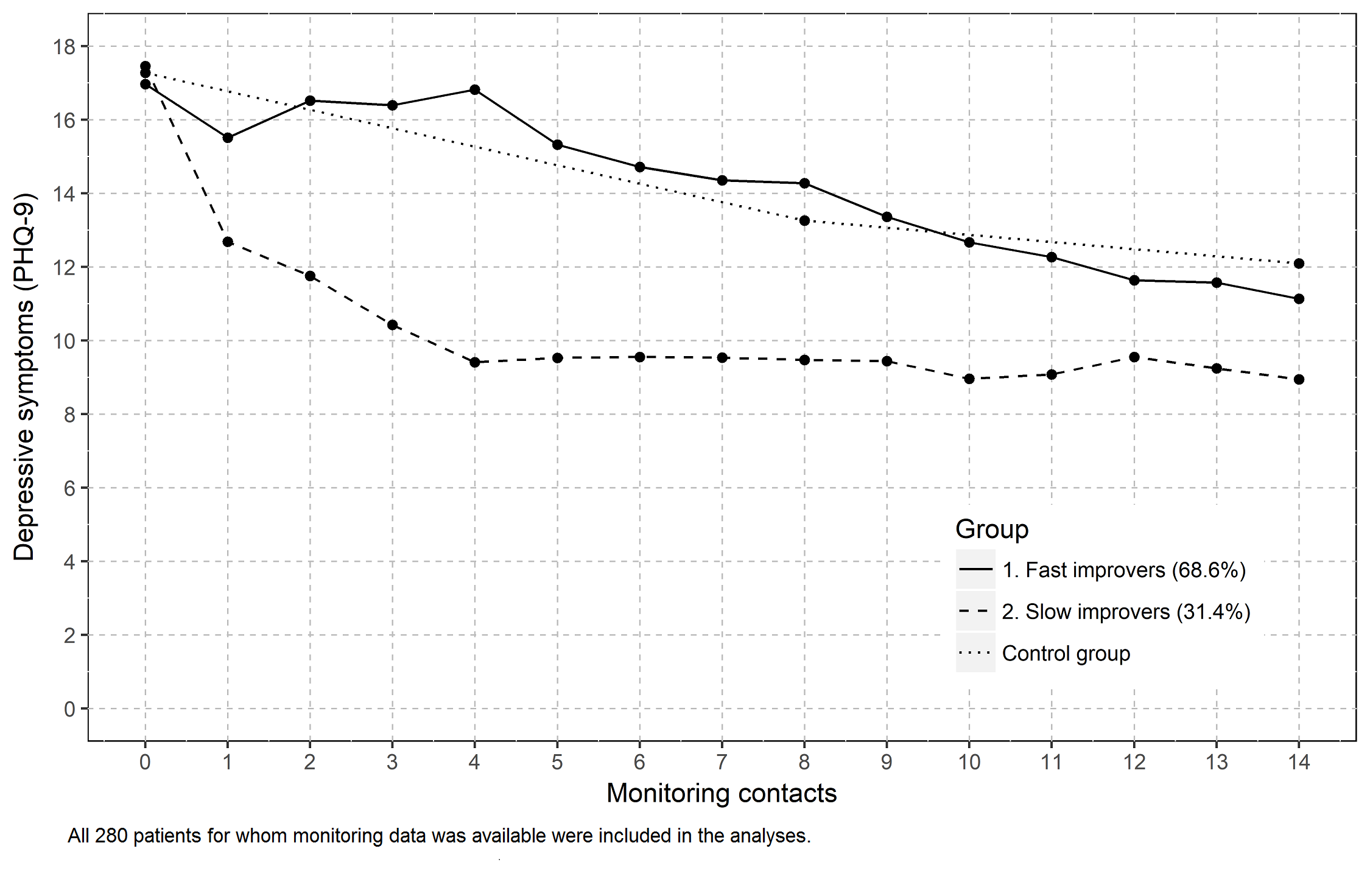

Supplement: S2 Fig — All 280 patients for whom monitoring data was available were included in the analyses. (TIF) [file pone.0202245.s002.tif]
